# Charcot–Marie–tooth disease causing mutation (p.R158H) in pyruvate dehydrogenase kinase 3 (PDK3) affects synaptic transmission, ATP production and causes neurodegeneration in a CMTX6 *C. elegans* model

**DOI:** 10.1093/hmg/ddab228

**Published:** 2021-08-13

**Authors:** Ramesh K Narayanan, Megan H Brewer, Gonzalo Perez-Siles, Melina Ellis, Carolyn Ly, Andrew Burgess, Brent Neumann, Garth A Nicholson, Steve Vucic, Marina L Kennerson

**Affiliations:** Northcott Neuroscience Laboratory, ANZAC Research Institute, Sydney, NSW 2139, Australia; Sydney Medical School, University of Sydney, Sydney, NSW 2006, Australia; Northcott Neuroscience Laboratory, ANZAC Research Institute, Sydney, NSW 2139, Australia; Northcott Neuroscience Laboratory, ANZAC Research Institute, Sydney, NSW 2139, Australia; Sydney Medical School, University of Sydney, Sydney, NSW 2006, Australia; Northcott Neuroscience Laboratory, ANZAC Research Institute, Sydney, NSW 2139, Australia; Northcott Neuroscience Laboratory, ANZAC Research Institute, Sydney, NSW 2139, Australia; Cell Division Laboratory, ANZAC Research Institute, Sydney, NSW 2139, Australia; Neuroscience Program, Monash Biomedicine Discovery Institute and Department of Anatomy and Developmental Biology, Monash University, Melbourne, Victoria 3800, Australia; Northcott Neuroscience Laboratory, ANZAC Research Institute, Sydney, NSW 2139, Australia; Sydney Medical School, University of Sydney, Sydney, NSW 2006, Australia; Molecular Medicine Laboratory, Concord General Repatriation Hospital, Sydney 2139, NSW, Australia; Concord Clinical School, University of Sydney, Sydney, NSW 2139, Australia; Northcott Neuroscience Laboratory, ANZAC Research Institute, Sydney, NSW 2139, Australia; Sydney Medical School, University of Sydney, Sydney, NSW 2006, Australia; Molecular Medicine Laboratory, Concord General Repatriation Hospital, Sydney 2139, NSW, Australia

## Abstract

Charcot–Marie-Tooth (CMT) is a commonly inherited, non-fatal neurodegenerative disorder that affects sensory and motor neurons in patients. More than 90 genes are known to cause axonal and demyelinating forms of CMT. The p.R158H mutation in the pyruvate dehydrogenase kinase 3 (*PDK3*) gene is the genetic cause for an X linked form of axonal CMT (CMTX6). *In vitro* studies using patient fibroblasts and iPSC-derived motor neurons have shown that this mutation causes deficits in energy metabolism and mitochondrial function. Animal models that recapitulate pathogenic *in vivo* events in patients are crucial for investigating mechanisms of axonal degeneration and developing therapies for CMT. We have developed a *C. elegans* model of CMTX6 by knocking-in the p.R158H mutation in *pdhk-2*, the ortholog of *PDK3*. In addition, we have developed animal models overexpressing the wild type and mutant form of human *PDK3* specifically in the GABAergic motor neurons of *C. elegans*. CMTX6 mutants generated in this study exhibit synaptic transmission deficits, locomotion defects and show signs of progressive neurodegeneration. Furthermore, the CMTX6 *in vivo* models display energy deficits that recapitulate the phenotype observed in patient fibroblasts and iPSC-derived motor neurons. Our CMTX6 animals represent the first *in vivo* model for this form of CMT and have provided novel insights into the cellular function and metabolic pathways perturbed by the p.R158H mutation, all the while closely replicating the clinical presentation observed in CMTX6 patients.

## Introduction

Charcot–Marie-Tooth (CMT) disease refers to a group of clinically and genetically heterogeneous disorders characterised by length-dependant axonal degeneration of motor and sensory neurons. CMT has a prevalence of 1 in 2500, making it the most commonly inherited neuropathy (1). CMT is a non-fatal disease leading to a poorly recognised economic health burden, in which patients suffer from lifelong disability and often require assisted living or full-time care (2). Gene mapping and cutting-edge genomic technologies have led to the discovery of over 1000 mutations in more than 90 genes that play a pathogenic role in CMT (3,4). Despite the advancement in our understanding of genetic causes of CMT, it is still not clear how these genetic mutations lead to cell specific (neuronal) and spatial-specific (peripheral) degeneration in patients. Consequently, there is still no specific treatment or cure available for CMT. Development of both *in vitro* and *in vivo* models, guided by the gene mutations of affected patients, will help replicate *in vivo* events in a preserved genetic background that causes the disease. These models will not only help our understanding of the cellular and biological pathways that lead to CMT but will serve as a useful platform for drug screening to identify compounds that can ameliorate disease progression or potentially cure CMT.

We previously reported a mutation in the pyruvate dehydrogenase kinase 3 (*PDK3*) gene (p.R158H) as the cause of an X-linked dominant form of CMT (CMTX6) in an Australian family (5). The same mutation was later reported in an unrelated X-linked CMT Korean family (6). The *PDK3* isoenzyme is a member of the pyruvate dehydrogenase kinase family expressed in the brain and spinal cord, that negatively regulates the pyruvate dehydrogenase complex (PDC) via reversible phosphorylation (5). PDC is a tightly regulated complex, that is comprised of pyruvate dehydrogenase (E1), dihydrolipoyl transacetylase (E2), dihydrolipoamide dehydrogenase (E3) and the E3 binding protein (E3BP) subunit. The complex catalyses the conversion of pyruvate to acetyl CoA, a precursor of the energy producing tricarboxylic acid cycle (TCA) cycle (7). The nuclear encoded PDK3 protein localises within the mitochondria, and negatively regulates the PDC complex by phosphorylating the pyruvate dehydrogenase E1 subunit. Restoration of PDC activity occurs when the E1 subunit is reversibly dephosphorylated by pyruvate dehydrogenase phosphatases (PDPs). Our initial *in vitro* enzyme studies showed the p.R158H *PDK3* mutation caused enzyme hyperactivity, which was predicted to cause hyperphosphorylation and subsequent reduction of PDC activity (5).

Using both CMTX6 patient fibroblasts, and iPSC-derived motor neurons we previously demonstrated that p.R158H-induced hyper-phosphorylation of pyruvate dehydrogenase (E1) reduces PDC activity (8,9). This led to significant reductions in ATP levels in both *in vitro* cellular models, thereby suggesting metabolic dysregulation due to the p.R158H mutation. Patient fibroblasts showed abnormal mitochondrial networks and mitochondrial trafficking was reduced in the axons of CMTX6 iPSC-derived motor neurons. Importantly, using a pan PDK inhibitor, dichloroacetate (DCA), the PDH hyperphosphorylation and metabolic defects seen in the patient cells were reversed, thus providing a proof of principle that CMTX6 phenotypes could be pharmacologically reverted (8,9).

In this study, we have used *C. elegans* (*C. elegans*) as a model for studying CMTX6. Of the 1000 gene mutations reported, we chose to model CMTX6 in the nematode as a complement to our CMTX6 patient *in vitro* cell models. We have generated CRISPR-Cas9 mediated knock-in animals with the arginine to histidine missense mutation at the conserved amino acid position 159 (R159H) in the orthologous *C. elegans* gene (*pdkh-2^R159H^*). In addition, we generated additional *C. elegans* models of CMTX6 by expressing human wild type (*hPDK3^WT^*) and mutant (*hPDK3^R158H^*) forms of PDK3 selectively in GABAergic motor neurons. We found that *C. elegans* models of CMTX6 showed: (i) reduced body width; (ii) axon-associated synaptic transmission deficits; (iii) defective stress response to paraquat induced oxidative stress; and (iv) reduced ATP levels as observed in our patient derived *in vitro* cell models. Furthermore, overexpression of human WT and p.R158H forms of *PDK3* specifically in the GABAergic motor neurons led to progressive neurodegeneration, resulting in locomotion defects. The *C. elegans* models generated in this study recapitulate various molecular phenotypes observed in both the CMTX6 fibroblasts and iPSC-derived motor neurons, and motor phenotypes observed in patients. Our *in vivo* models will help advance an understanding of CMTX6 pathophysiology and complement our patient derived *in vitro* models in future research.

## Results

### Generation of knock-in and overexpression models of *C. elegans* for studying CMTX6

**Figure 1:**
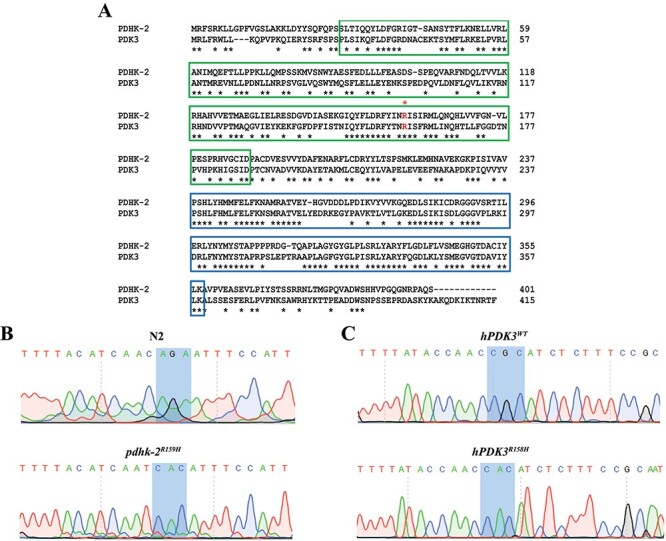
**Knocking-in and overexpression of CMTX6 causing mutation in *C. elegans*.** (A) Alignment of full-length amino acid sequences of human PDK3 (415 amino acids) and the *C. elegans* ortholog, PDHK-2 (401 amino acids) using ClustalW. Highlighted boxed regions indicate conserved protein domains [Green: BCDHK_Adom3 (Mitochondrial branched-chain alpha-ketoacid dehydrogenase kinase; Blue: HTPase_PDK-like (Histidine kinase-like ATPase domain of pyruvate dehydrogenase kinase)]. The conserved amino acid position in human where the reported mutation is present is highlighted by an FX1. Conserved amino acid residues between PDK3 and PDHK-2 are indicated by ^*^. (B) Sequence trace of *pdhk-2* from N2 and knock-in mutant generated using CRISPR-Cas9. The highlighted region indicates the nucleotide changes used to introduce histidine at amino acid residue 159 in the knock-in model. More information on CRISPR modification is available in the supplementary information. (C) Sequence trace confirming the human wild type and mutant *PDK3*. Highlighted region shows the codon change from CGC (encoding an arginine in wild type *hPDK3*) to CAC (encoding a Histidine in the mutant p.R158H *PDK3*).

CMTX6 fibroblasts, patient-derived iPSCs and iPSC-derived motor neurons have provided insights into altered metabolism, mitochondrial network and trafficking abnormalities of CMTX6 resulting from the *PDK3* p.R158H mutation. We have used the nematode *C. elegans* as an *in vivo* model for studying CMTX6. The worm orthologue of human *PDK3* (*pdhk-2*) is evolutionarily conserved (Fig. 1A). Two different approaches for generating transgenic CMTX6 animals were used. Firstly, we generated a knock-in model utilising CRIPSR-Cas9 technology to knock in the CMTX6 missense R158H mutation at the conserved amino acid position R159 in *C. elegans*, highlighted by the asterisk in Fig. 1A. Secondly, overexpression models of CMTX6 were generated by expressing the WT and R158H forms of human *PDK3* under control of the *unc-25* promoter, which drives gene expression selectively in the GABAergic motor neurons (10). Although human motor neurons are predominantly cholinergic in nature, in *C. elegans*, both GABAergic and cholinergic neurons are involved in animal locomotion. In addition, GABAergic neurons innervate body wall muscle in *C. elegans* thus allowing us to study the effect of CMTX6 causing mutation on neuromuscular junction (NMJ), a site affected in several forms of CMT (11,12). The human transgenes were microinjected (at a concentration of 10 ng/μL) into an animal carrying an integrated *oxIs12* allele, which facilitates the visualisation of GFP expression in the neurons of live animals, under the control of *unc-47* promoter. Sanger sequencing confirmed the genotype of the knock-in mutant strain (*pdhk-2^R159H^*), which showed nucleotide changes (AGA to CAC) that introduce the mutant codon (Fig. 1B). CMTX6 overexpression models were also Sanger sequenced to confirm the wild type and p.R158H human cDNA sequence (Fig. 1C).

### CMTX6 *in vivo* models show reduced body width

**Figure 2:**
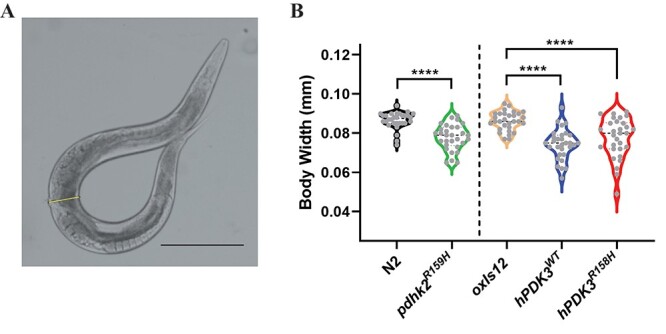
**The knock in and overexpression of the *PDK3* mutation causing CMTX6 affects the body width in *C. elegans*.** Sample image of an N2 animal and the methodology used for measuring body width (A) using Image J software. The squares indicate the location along the animal body selected to construct the yellow line, which represents the width (from the end of the vulva on the ventral side to the dorsal side) of the animal. The length of the yellow line has been calibrated within the software according to the reference scale bar. Scale bar is 0.25 mm. Violin plots of body width (B) observed when comparing CMTX6 animals and their respective controls. Black dotted line represents median value of the data sets and grey dots represents measured data points for individual animals. The number of animals used for the body width measurements: N2 (n = 30), *pdhk-2^R159H^* (n = 30), *oxIs12* (n = 30), *hPDK3^WT^* (n = 30) and *hPDK3^R158H^* (n = 30). ^*^^*^^*^^*^ adjusted p-value < 0.0001.

The cell size rather than cell number regulates body size in *C. elegans*. The nervous system along with the neuroendocrine system plays a role in regulating cell size in *C. elegans* (13). In addition, altered sensory perception, due to defective kinase activity leads to reduced growth in *C. elegans* (14). Taken together, a dysfunctional nervous system and aberrant kinase activity, in our case *PDK3,* might lead to changes in the worm’s body size (width). Measurement of these features in animals 48 h post-larval stage 4 demonstrated a statistically significant reduction in body width in the CMTX6 models (Fig. 2). The mean body width (mm ± SEM) analysis in these strains identified statistically significant differences between controls [N2 animals (0.08650 ± 0.00105) and *oxIs12* (0.086 ± 0.00087)] and the CMTX6 models [*pdhk-2^R159H^* = 0.07730 ± 0.00164, *hPDK3^WT^* = 0.07408 ± 0.00169 and *hPDK3^R158H^* = 0.07836 ± 0.00186]. *hPDK3^R158H^* animals showed a slight increase in mean body width when compared to *hPDK3^WT^*. The difference observed between the overexpression animal models was not significant [*hPDK3^WT^* vs *hPDK3^R158H^*: body width adjusted p-value = 0.2988].

### CMTX6 mutation causes pre-synaptic (neuronal) transmission deficits

**Figure 3:**
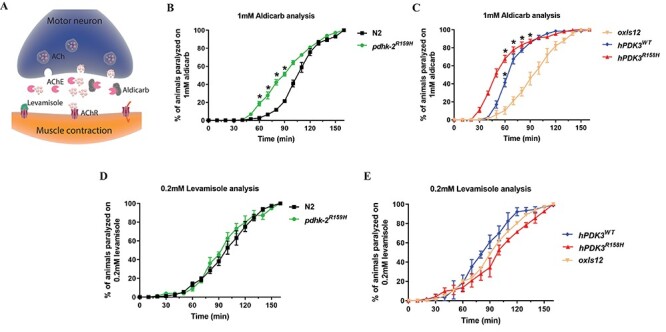
**Synaptic transmission defect in CMTX6 models of *C. elegans*.** (A) Illustration of *C. elegans* neuromuscular junction and the actions of aldicarb and levamisole at the synapse. Aldicarb is a nematicide that cleaves acetylcholinesterase, an enzyme that regulates acetylcholine levels, resulting in paralysis and eventually death in worms. Aldicarb identifies potential deficits associated with synaptic transmission in *C. elegans*. Levamisole is an acetylcholine receptor blocker and allows identifying the role of pre-synaptic (neuron) or/and post-synaptic (muscle) compartment in the aldicarb induced phenotype. (B and C) *C. elegans* strains were treated with 1 mM aldicarb. CMTX6 animals showed significant sensitivity to aldicarb when compared to their respective controls (N2 and *oxIs12*), which indicates deficits in synaptic transmission. *C. elegans* overexpressing human mutant *PDK3* (*hPDK3^R158H^*) showed the highest sensitivity to aldicarb followed by those expressing human wild type *PDK3* (*hPDK3^WT^*) and knock-in mutant (*pdhk-2^R159H^*). 20–35 animals were used in one biological replicate per genotype with the numbers of biological replicates used are as follows: N2 = 12; *oxIs12* = 3; *pdhk-2^R159H^* = 9, *hPDK3^WT^* = 3, and *hPDK3^R158H^* = 10. The mean ± SEM is presented. ^*^ p-value < 0.05, two-tailed unpaired t-test. (D and E) *C. elegans* strains treated with 0.2 mM levamisole. No difference was observed in the time at which the CMTX6 animals paralysed in response to levamisole treatment when compared to controls, suggesting the involvement of the pre-synaptic compartment (neuron) in the aldicarb induced phenotype observed in the CMTX6 mutants. 20–35 animals per biological replicate per genotype with the numbers of biological replicates used are as follows: N2 = 6; oxIs12 = 3; *pdhk-2^R159H^* = 3, *hPDK3^WT^* = 3 and *hPDK3^R158H^* = 3. Error bars indicate mean ± SEM.

Biochemical assays using anti-helminthic drugs aldicarb and levamisole in *C. elegans* are widely used for identifying mutants with synaptic transmission defects (15). In these assays, increased sensitivity to these compounds is associated with pre-synaptic and/or post-synaptic defects (Fig. 3A). The acetylcholinesterase enzyme regulates acetylcholine levels in the synaptic cleft at the neuromuscular junction (NMJ). Aldicarb, an acetylcholinesterase inhibitor leads to an increase in acetylcholine levels in the synapse resulting in paralysis and eventual death in *C. elegans*. CMTX6 animals displayed a hypersensitive phenotype when exposed to 1 mM aldicarb when compared to their respective controls (Fig. 3B and C). In this experiment, the time at which 50% of the assayed animals showed paralysis was 110 min for the N2 and *oxIs12*, significantly longer than the time observed for CMTX6 animals. The knock-in *pdhk-2^R159H^* animals showed 50% paralysis at 90 min. The overexpression animal models of CMTX6 showed the highest sensitivity to aldicarb exposure, with the mutant form of *PDK3* (*hPDK3^R158H^*) being the most sensitive strain (45 min to observe paralysis in 50% of the worm population, versus 60 min for those expressing the wild type human *PDK3*, *hPDK3^WT^*). Levamisole is an acetylcholine receptor antagonist that causes paralysis in *C. elegans* and is used to define if the pre-synaptic (neuron) and/or post-synaptic compartment (muscle) is contributing to the aldicarb phenotype. Time-course paralysis assays of the CMTX6 animals using levamisole indicated that there was no significant difference in the rate of paralysis of wild type and transgenic animals, thereby excluding involvement of the post-synaptic component (muscle) in the aldicarb phenotype (Fig. 3D and E). Taken together, our biochemical assays indicate that the CMTX6 mutants are defective in synaptic transmission due to pre-synaptic (neuron) defects. These findings correlate with the axonal presentation of CMTX6.

### The *PDK3* mutation does not affect mitochondrial number in *C. elegans* but increases susceptibility to oxidative stress

**Figure 4:**
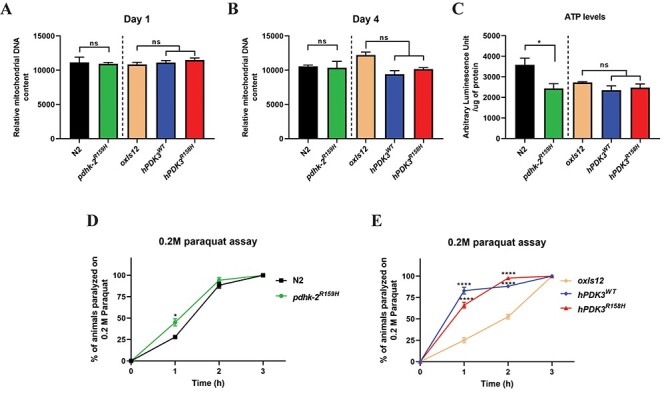
**CMTX6 models of *C. elegans* recapitulate ATP deficits observed in CMTX6 patients and are susceptible to paraquat induced oxidative stress.** PCR based mitochondrial DNA copy number quantification shows that there is no change in mitochondrial DNA copy number at day 1 (A) and day 4 (B) old animals. (C) ATP production in day 1 old *C. elegans* strains. ATP production is significantly reduced in knock-in *pdhk-2^R159H^* animals. There was no significant difference in ATP production in overexpression CMTX6 animal lysates when compared to *oxIs12* animals. N = 1500 to 3000 age synchronised day 1 old animals per replicate per genotype. A total of three biological replicates were used for the ATP production assay. Average luminescence of ATP normalised to 1 μg of total protein is presented. ^*^ adjusted p-value < 0.05. (D and E) 0.2 M paraquat assay. 1 h post treatment, CMTX6 animals showed increased sensitivity to oxidative stress with knock-in mutants showing a significant increase in the percentage of mortality while the overexpression animal models of CMTX6 displayed mortality greater than 50%. N = 6 animals per genotype per replicate was used for mitochondrial DNA copy number quantification. A total of 3 biological replicates were used. For paraquat assay N = 25 to 38 animals per genotype per replicate was used with a total 3 biological replicates used in the experiment. ^*^ adjusted p-value = 0.0353 and ^*^^*^^*^^*^ adjusted p-value < 0.0001. Error bars indicate ± SEM.

CMTX6 patient derived *in vitro* cell models have shown altered mitochondrial networks, reduced ATP production and aberrant mitochondrial trafficking (8,9). To understand the impact of CMTX6 causing *PDK3* mutation on mitochondrial replication *in vivo*, we adapted a previously established non-quantitative PCR method to assess the mitochondrial copy number of the CMTX6 animals used in this study (16). We observed no significant difference in the mitochondrial DNA copy number between day 1 and day 4 old CMTX6 animals and their age matched controls (Fig. 4A and B). *In vitro* cellular models demonstrated that the p.R158H mutation caused a reduction in ATP levels. Therefore, we assessed the production of ATP in day 1 old animals. We observed a significant reduction in free cellular ATP levels in the knock-in *pdhk-2^R159H^* animals (Fig. 4C). There was no significant difference in the ATP levels between the overexpression models of CMTX6 and *oxIs12* animals.

Oxidative stress has previously been implicated in CMT pathogenesis (17–19). To determine if the *PDK3* p.R158H mutation leads to defective oxidative stress, we exposed CMTX6 animals to 0.2 M paraquat, which has been widely used to generate oxidative stress conditions in *C. elegans* (20). Like the anti-helminthic chemicals, paraquat treatment leads to paralysis and death. As such, we scored the animals for paralysis every 1 h for a period of 3 h. After 1 h of paraquat treatment, 27% of the N2 and 25% of the *oxIs12* animals displayed paralysis while the CMTX6 animals [*pdhk-2^R159H^* (45%), *hPDK3^WT^* (83%), *hPDK3^R158H^* (66%)] showed significantly increased paralysis when compared to their respective controls (Fig. 4D and E). These results demonstrate that the CMTX6 animals are hypersensitive to oxidative stress.

### Overexpression of the wild type and mutant human *PDK3* in GABAergic motor neurons leads to neurodegeneration and affects locomotion in *C. elegans*

**Figure 5:**
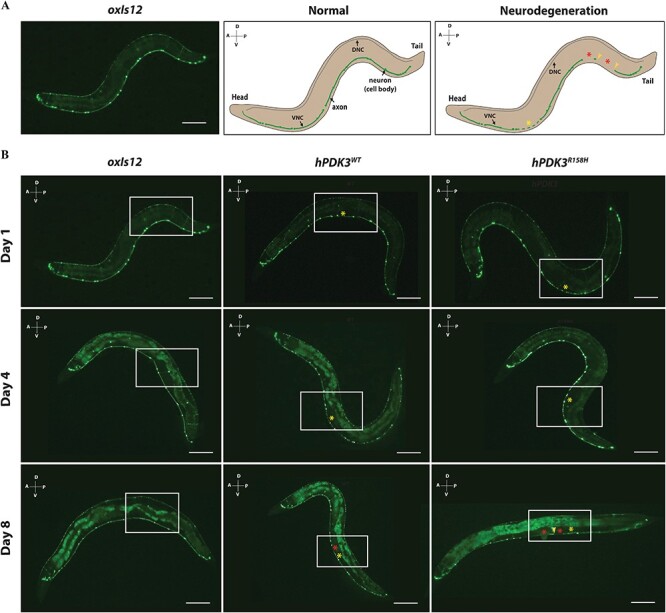
**Overexpression of human wild type and mutant *PDK3* in motor neurons leads to neurodegeneration in *C. elegans*.** (A) Neurodegeneration scoring paradigm. Representative image of day 1 old *oxIs12* animals and schematic illustrating normal and neurodegenerative phenotypes. The morphology of the ventral nerve cord (VNC) of *C. elegans* was analysed for assessing the impact of over expression of wild type and mutant PDK3 on neuron morphology. The dorsal nerve cord (DNC) was not included in scoring due to the reduced GFP expression. In the normal animal, the axon of the ventral nerve cord is intact, which is indicated by continuous GFP expression, with all the neurons (cell bodies) still visible. The presence of blebbing or beading of the axons as indicated by yellow asterisk FX2 and/or the complete loss of axon (indicated by red asterisk FX3) and/or neuron (yellow arrowhead) in the VNC of *C. elegans* is scored as neurodegeneration phenotype. A-Anterior, P-posterior, D-Dorsal and V-ventral; Scale bar = 0.3 mm. (B) Representative images of day 1, day 4 and day 8 old wild type (*oxIs12*) and overexpression CMTX6 animals (*hPDK3^WT^* and *hPDK3^R158H^*). Boxed region highlights the difference between *oxIs12* and CMTX6 overexpression animals. CMTX6 over expression mutants show signs of axon degeneration at day 1. Yellow asterisk FX4 indicate region of the axon showing blebbing or beading (degeneration). There was no sign of complete loss of axon or neuron (cell body) in day 1 old animals. Regions of axon loss (loss or interruption of GFP signal along the VNC is indicated by the red asterisk FX5. CMTX6 overexpression animals show increased signs of axon degeneration characterised by beading or blebbing and loss of GFP signal at day 4. Axon degeneration is more severe in the overexpression CMTX6 animals at day 8 with *hPDK3^R158H^* animals showing complete loss of axon (indicated by red asterisk FX6) and neuron cell body (yellow arrowhead). Number of animals used for live imaging are as follows: Day 1–31 *oxIs12*, 26 *hPDK3^WT^* and 27 *hPDK3^R158H^* animals; Day 8–46 oxIs12, 38 *hPDK3^WT^* and 45 *hPDK3^R158H^* animals; A-Anterior, P-posterior, D-Dorsal and V-ventral; Scale bar—0.3 mm.

**Figure 6:**
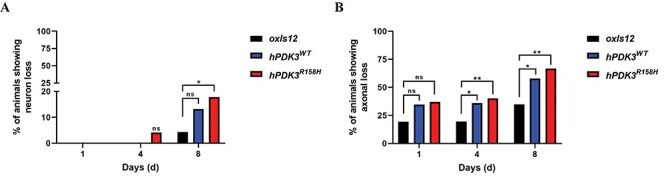
**Quantification of neuron and axon loss in overexpression CMTX6 animals reveals progressive neurodegeneration.** (A) No neuron loss was observed in day 1 old CMTX6 and control animals. *hPDK3^R158H^* showed signs of neuron loss at day 4 and with the number of overexpression CMTX6 animals exhibiting neuron loss significantly higher than the *oxIs12* animals at day 8. (B) Axon degeneration is more prominent in the overexpression animal models at day 4, with day 8 old overexpression CMTX6 animals showing complete loss of axon and several neurons. Animals used for live imaging are as follows: day 4 *oxIs12* (n = 87), *hPDK3^WT^* (n = 98) and *hPDK3^R158H^* (n = 97). There is no error bar because of the statistical test used for analysis. A chi-square test was used to compare categorical data (presence of wild type neuron/axon or axon/neuron loss). Neuron loss: ^*^ p-value = 0.0406; Axon loss: ^*^ p-value < 0.05, ^*^^*^p-value = 0.0024, ns—not significant.

The *PDK3* p.R158H mutation leads to progressive degeneration of peripheral nerves in CMTX6 patients (5,6). *C. elegans* overexpression models have previously been used to study neurodegeneration in CMT (21). In this study, we generated overexpression models of CMTX6 to observe the impact of human wild type and mutant *PDK3* on motor neuron degeneration *in vivo*. To study the progression of the motor neuron degeneration, we analysed age-synchronised animals (1, 4 and 8 day old). Animals carrying the *oxIs12[unc-47::GFP]* transgene allow the GABAergic neurons to be visualised with GFP and were used for generating overexpression CMTX6 models. CMTX6 overexpression mutants displayed both axonal degeneration and neuron loss (Fig. 5B). While the neuron loss in the *hPDK3^WT^* model was only observed in day 8 old animals (13% of screened worms), we observed that 4% and 17% of the mutant *hPDK3^R158H^* animals showed signs of neuron loss at days 4 and 8, respectively (Fig. 6A). Importantly, neuron loss was not observed in controls at day 1 and day 4, and only 4% of control animals showed neuron loss at day 8. Interestingly, our overexpression CMTX6 animal models showed signs of axon loss (blebbing or beading phenotype or loss of GFP signal along ventral nerve cord (VNC) at day 1 (34% *hPDK3^WT^* and 37% *hPDK3^R158H^*) and an increase in the percentage of animals affected at day 4 (35% *hPDK3^WT^* and 40% *hPDK3^R158H^*) and day 8 (57% *hPDK3^WT^* and 66% *hPDK3^R158H^*) (Fig. 6B). These events were significantly more frequent in the *hPDK3^WT^* and *hPDK3^R158H^* strains than in control animals at days 4 and 8. The neurodegenerative phenotype observed clearly demonstrate that axon loss precedes neuron loss in the CMTX6 animals, indicating the vulnerability of axons to *PDK3* overexpression. In addition, our data shows the progressive nature of axonal degeneration in CMTX6 animals, which reflects the age-dependent severity observed in CMTX6 patients.

**Figure 7:**
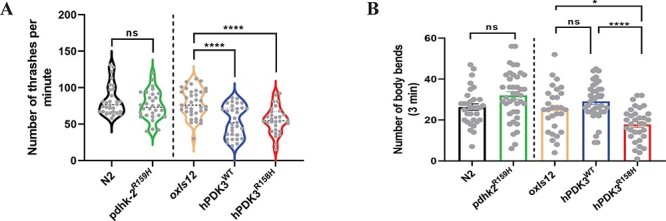
**Overexpression animal models of CMTX6 show locomotion defects.** (A) Body thrash quantification of CMTX6 animals. There was no significant difference in the number of body thrashes for the knock-in animals when compared to N2. CMTX6 animals expressing the human *PDK3* gene (*hPDK3^WT^* and *hPDK3^R158H^*) in the GABAergic motor neurons, showed significant reduction in the number of body thrashes when compared to *oxIs12* animals. Number of animals used for thrashing assay: n = 30 for N2, *pdhk-2^R159H^* and *hPDK3^R158H^*; *hPDK3^WT^* (31); *oxIs12* (37). ^*^^*^^*^^*^ adjusted p-value < 0.0001, ns—not significant. (B) Quantification of body bends in the *C. elegans* mutants of CMTX6. *pdhk-2^R159H^* and *hPDK3^WT^* animal models of CMTX6 showed no difference in the number of body bends when compared to their respective controls. *hPDK3^R158H^* worms showed a reduction in the number of body bends when compared to *hPDK3^WT^* and *oxIs12* animals. Grey spheres indicate data from individual animals. n = 32 N2, 40 *pdhk-2^R159H^*, 29 *oxIs12*, 24 *hPDK3^WT^* and 33 *hPDK3^R158H^* for the body bend experiment. ^*^ adjusted p-value = 0.031, ^*^^*^^*^^*^ adjusted p-value < 0.0001, ns—not significant. Error bars indicate ± SEM.

Quantifying the axon and neuronal loss in the CMTX6 animals clearly showed that overexpression of the wild type and the mutant form of human *PDK3* is highly toxic to neurons and resulted in axonal degeneration. Therefore, we studied the impact of neurodegeneration on animal locomotion. We analysed both the body thrash and body bend behavioural phenotypes for the CMTX6 animals, which are widely used assays to study locomotion in *C. elegans* models of neurodegeneration (22,23). Based on our observations in Fig. 6A and B, we chose day 4 old animals for the locomotion behaviour assay as they showed clear signs for the onset of neuronal degeneration but with most of the axons still intact and minimum to no neuron loss. The *pdhk-2^R159H^* knock-in mutants showed no significant change in body thrashes or body bends when compared to N2 (Fig. 7A and B). In contrast, *hPDK3^R158H^* animals displayed reduced locomotion ability in both assays (~33% and 30% reduction in thrashing and body bends respectively). Furthermore, *hPDK3^R158H^* animals demonstrated a significant reduction in the number of body bends when compared to *hPDK3^WT^* animals. CMTX6 animals with overexpression of the human wild type *PDK3* in the motor neurons (*hPDK3^WT^*) showed a significant reduction only in the number of thrashes (Fig. 7A), while body bending was not affected (Fig. 7B). Thus, the overexpression of PDK3 led to movement defects in our *C. elegans* models, with the *hPDK3^R158H^* animals demonstrating more pronounced deficits.

## Discussion

CMT is a non-fatal neurodegenerative disorder that affects the motor and sensory nerves resulting in lifelong disability for patients. Identification of several CMT causing gene mutations has improved the diagnosis of CMT and led to the development of animal models. Numerous rodent models have shown symptoms similar to CMT, providing crucial insights into disease progression, as well as identifying drug candidates in preclinical studies; but this has failed to translate in the clinical setting (24). Since reporting the CMTX6 *PDK3* gene mutation, our laboratory has developed *in vitro* cellular models, including patient fibroblasts and iPSC-derived motor neurons from CMTX6 patients, which have provided crucial insights into CMTX6 pathogenesis (8,9). Patient iPSC-derived motor neurons provide a relevant neuronal tissue for studying the disease aetiology as well as identifying potential treatment compounds in pharmacological studies. However, these models lack the systems biology context that an *in vivo* animal model presents with an intact nervous system and associated support cells. Developing an *in vivo* model was therefore a necessary step towards understanding the role of the p.R158H mutation in CMTX6 and the effect of the mutation on locomotion and age-related neurodegeneration in a live organism, both of which cannot be answered using *in vitro* cellular models. In this study, we have generated and characterised an invertebrate model of CMTX6 using *C. elegans*. *C. elegans* has been an ideal model organism for understanding nervous system development and function, synaptic transmission, axon regeneration and for studying several neurodegenerative diseases including Alzheimer’s (25,26), Parkinson’s (27–29), ALS (23,30,31), CMT (12,21,22,32) and other neurodegenerative disorders (33). This study has used both knock-in and overexpression models to understand CMTX6 disease aetiology caused by the *PDK3* p.R158H mutation. Our data shows that the *PDK3* mutation leads to nervous system dysfunction and neurodegeneration resulting in locomotion deficits in *C. elegans*. In addition, our *in vivo* knock-in CMTX6 model recapitulates the energy deficits observed in CMTX6 patient fibroblasts and iPSC-derived motor neurons.

The worm *PDK3* orthologue, *pdhk-2* has been shown to play a major role in the regulation of energy metabolism (Personal communication: Prof. Young-ki Paik, January 2021) and in fat metabolism especially under adverse conditions (34). Furthermore, *pdhk-2* has previously been shown to regulate oxidative stress responses in *C. elegans* (35). The knock-in CMTX6 model (*pdhk-2^R159H^*) generated in this study reflects the genetic background of CMTX6 patients and will help understand the metabolic deficits associated with p.R158H mutation in CMTX6. In addition, we generated transgenic *C. elegans* models overexpressing wild type and p.R158H *PDK3* in the GABAergic motor neurons under the control of the *unc-25* promoter. Overexpression of the *C. elegans hars-1* gene (the ortholog of human *HARS* gene) carrying the CMT causing p.D364Y mutation, under the control of *unc-25* promoter specifically in the GABAergic motor neurons led to degeneration of axonal commissures (21). Similarly, the overexpression CMTX6 models generated in this study demonstrated axon and neuron loss and dysregulated fat storage (Supplementary Material, Fig. S2).

*C. elegans* transgenic mutants generated in this study displayed reduced body width (Fig. 2B). Nervous and neuroendocrine systems play major roles in animal development in *C. elegans*. In an Alzheimer’s disease model of *C. elegans*, overexpression of APL-1, the *C. elegans* ortholog for human Amyloid Precursor Protein (APP), led to reduced body length and resulted in locomotion defects (36). Knock-in and overexpression models of Alzheimer’s disease generated displayed reduced body length and width when compared to N2 animals. Furthermore, *apl-1* animals showed hypersensitivity to aldicarb. Pleiotropic phenotypes such as animal viability, sensitivity/resistance to pharmacological assays along with neuronal deficits associated with motor or sensory neurons are used as readouts for neuronal dysfunction/neurodegeneration in *C. elegans* (33). Similarly, CMTX6 animals generated in this study exhibited altered body morphology (Fig. 2B) and an aldicarb hypersensitivity phenotype (Fig. 3B and C) indicating synaptic transmission defect. The knock-in and overexpression models displayed a variable hypersensitivity phenotype in the presence of aldicarb. Given that the p.R158H mutation leads to increased kinase activity in CMTX6 patients, it is not surprising that overexpression of *PDK3* (wild type or mutant) in *C. elegans* results in a severe synaptic transmission defect when compared to the knock-in mutant animals. However, it is clear from our studies that *PDK3* plays a major role in synaptic transmission and that hyperactivity of PDK3 results in altered synaptic transmission *in vivo*. The p.R158H mutation in *PDK3* causes an axonal form of CMT. It was therefore important to assess the role that post-synaptic (muscle) components contribute to the hypersensitive phenotype observed upon exposure to aldicarb. In the presence of levamisole, an acetylcholine receptor antagonist, the CMTX6 mutants behaved the same as the control animals, thereby stipulating that the aldicarb phenotype results from pre-synaptic (axonal) associated defects (Fig. 3D and E).

The *PDK3* p.R158H mutation leads to altered mitochondrial matrix morphology in CMTX6 patient fibroblasts (9). Disruption of the mitochondrial network has been shown to impact mitochondrial function leading to reduced ATP output (37), a common theme in CMT and other neurodegenerative diseases (38–40). We also demonstrated altered energy metabolism in our CMTX6 *in vitro* cell models. However, it was unresolved if mutant *PDK3* was impacting on the number of viable mitochondria. To address this, we used a well-established PCR method to quantify the mitochondrial DNA copy number in our CMTX6 mutant strains. We found no significant changes in the mitochondrial number in the CMTX6 animals at day 1 and day 4 when compared to controls (Fig. 4A and B). Mitochondrial DNA copy number of whole animals, rather than in specific compartments such as neurons were assessed in this study. Thus, analysis of mitochondrial network in the affected neuronal populations may be useful in future studies. ATP quantification of day 1 old animals showed a significant reduction in ATP levels in our knock-in CMTX6 animals (Fig. 4C), thereby recapitulating the ATP phenotype of patient derived fibroblasts and iPSC-derived motor neurons. However, the ATP levels of the overexpression CMTX6 animals remained unchanged. Perhaps, the spatial specific overexpression of PDK3 has little to no impact on overall ATP production. Taken together, our results show that CMTX6 causing mutation has no effect on the mitochondrial number but knocking in the p.R158H CMTX6 mutation reduced ATP production. One approach to assess the effect of abnormal mitochondrial function is to use oxidative stress as a phenotypic readout, as this process is associated with various neurodegenerative diseases (41). Paraquat induces oxidative stress by increasing mitochondrial superoxide levels (42). Following one hour treatment with paraquat, a significantly higher percentage of CMTX6 animals showed signs of paralysis when compared to controls indicating oxidative stress as a potential disease mechanism in CMTX6 (Fig. 4D and E). Interestingly, 2-hour post paraquat treatment, *oxIs12* animals showed increase resistance when compared to N2 animals, which might be attributed to different stock solutions used for the assays. However, like the pharmacological assays, the overexpression CMTX6 animals exhibited a dramatic increase in paralysis (> 65%) when compared to the knock-in mutants (25%), indicating that *PDK3* overexpression leads to increased sensitivity to oxidative stress in *C. elegans*.

Strains carrying the *oxIs12* allele, which allows the visualisation of GABAergic neurons (43) have previously been used to visualise and quantify neurodegeneration in a CMT model of *C. elegans* (44). CMTX6 overexpression models show signs of neurodegeneration (Fig. 5B) that progressively becomes worse as the animals age (Fig. 5 and 6). Older males in the Korean and Australian CMTX6 families showed severe disability when compared to younger patients, indicating the progressive nature of axonal degeneration in CMTX6 (5,6). Neurodegeneration in our CMTX6 animals is progressive, thus closely replicating the clinical presentation of CMTX6 patients. Importantly, axon loss preceded neuron loss in our CMTX6 *C. elegans* model, indicating that axonal degeneration occurs before eventual neuron loss. Given that axonal degeneration is a significant pathological event in CMT, animal models generated in this study provides a platform for studying molecular events that lead to axon vulnerability in CMTX6. Furthermore, several neurodegenerative disorders such as Alzheimer’s, Parkinson’s, Motor Neuron Disease and other forms of CMT are characterised by progressive neurodegeneration (45).

To assess the impact of neurodegeneration on animal locomotion, we performed well established locomotion assays to study animal movement in liquid (thrashing) and on solid medium (body bend). *C. elegans* recruits different subsets of neurons and employs unique motor paradigms for moving in liquid and solid medium and therefore allows us study animal behaviour at a neuronal circuit level (46,47). The body thrashing assay showed that the knock-in animals behaved very similar to N2, whereas the overexpression animal models showed reduced locomotion (~ 30%) in the liquid medium when compared to *oxIs12* animals (Fig. 7A). On solid medium, both *pdhk-2^R159H^* and *hPDK3^WT^* animals behaved similar to their respective controls. However, *hPDK3^R158H^* animals showed a 30% reduction in the number of body bends when compared to *oxIs12* animals and a 40% reduction when compared to *hPDK3^WT^* animals (Fig. 7B). *pdhk-2^R159H^* animals did not exhibit any locomotion defect and is likely due to *pdhk-2* being absent in the ventral nerve cord of *C. elegans*. Taken together our locomotion assays show that the overexpression of mutant *PDK3* causes locomotion deficits *in vivo*. We identified day 4 as a critical time point, when CMTX6 animals begin to exhibit axonal degeneration and locomotion defects with neuronal cell bodies still intact. Gene dosage plays a major role in the manifestation of the severity of the disease in CMTX6. Although displaying stable transgene inheritance, controlling for gene dosage in our extra-chromosomal arrays is challenging. Generation of single-copy insertion strains will be beneficial for further understanding the role of gene dosage pathogenesis in male and female CMTX6 patients and will greatly aid in future pharmacological intervention studies.

In conclusion, we have generated and characterised *in vivo* models for studying CMTX6 using *C. elegans*. By using knock-in and over expression models we were able to tease out mechanisms the mutation affects, such as synaptic transmission. The models recapitulate the energy deficits observed in CMTX6 patient derived *in vitro* cell models, providing further evidence on the importance of energy production and metabolism in axonal degeneration. We have demonstrated that cell-specific overexpression of wild type and mutant *PDK3* in GABAergic neurons leads to progressive neurodegeneration in *C. elegans* resulting in locomotion problems. The CMTX6 models of *C. elegans* generated in this study compliments our previously established CMTX6 cellular models and will serve as an *in vivo* platform for screening drugs that might help stop the disease progression in CMTX6 patients. Given that energy deficits are associated with other forms of CMT and various other neurodegenerative diseases, pharmacological intervention studies to reverse energy deficits in our CMTX6 model may have a significant impact on the study of neurodegenerative disorders.

## Materials and Methods

### *C. elegans* strains

*C. elegans* used in this study were maintained on nematode growth medium (NGM) seeded with OP50 at 22°C as previously described (48). The Bristol N2 strain obtained from the Caenorhabditis Genetics Centre (CGC) was used as the background for generation of knock-in *pdhk-2^R159H^* mutants and therefore used as the wildtype control for the knock-in animals. Generation of knock-in mutants was outsourced to SunyBiotech Pty Ltd The EG1285 strain carrying *oxIs12* allele [*unc-47p::GFP + lin-15(+)*], which allows the visualisation of GABAergic neurons with GFP, was used for the generation of overexpression animal models and as a control in experiments for overexpression animal models. Transgenic overexpression animal models were generated by expressing the wild type or p.R158H forms of the human *PDK3* gene under the control of *unc-25* promoter, which drives the expression of the human transgene in GABAergic motor neurons. Generation of knock-in and overexpression models of *C. elegans* can be found in the supplementary methods. The list of animals and associated genotypes used in this study are listed in Table 1.

**Table 1:** List of *C. elegans* strains used in this study

Abbreviation	Strain	Genotype	WormBase Acc. No.
	N2	Wild type, Bristol strain	
*oxIs12*	EG1285	*oxIs12[unc-47p::GFP + lin-15(+)]*	
*pdhk-2^R159H^*	PHX932	*pdhk-2*(*syb932*[R159H])	WBStrain00048670
*hPDK3^WT^*	MHB1	*nnaEx1[unc-25p::PDK3(+)]; oxIs12[unc-47p::GFP + lin-15(+)]*	WBStrain00048671
*hPDK3^R158H^*	MHB2	*nnaEx2[unc-25p::PDK3(R158H)]; oxIs12[unc-47p::GFP + lin-15(+)]*	WBStrain00048672

### Single worm PCR

The *C. elegans* Lysis/DNA extraction kit (InVivo Biosystems) was used for preparing worm lysates for PCR analysis. A single worm was placed in 5x Worm Lysis buffer A (2 μL), 10x Worm Lysis Buffer B (2 μL) and RNAse/DNAse free water (7 μL) (Invitrogen). Single worm lysates were prepared by incubating the reaction mix for 5 min at 75°C followed by 10 min at 95°C. Following incubation, RNAse/DNAse free water (90 μL) was added to make the lysate volume up to 100 μL. All PCR amplification reactions except for mitochondrial DNA copy number quantification was carried out using PCR Master Mix from In Vivo Biosystems following manufacturer’s instruction. Amplification and genotyping of worm *pdhk-2* gene was carried out using the following primers: Forward: 5’-GACACGCACATGTCGTCGAAA-3′, Reverse 5’-CGGACAGCGAAGAATAGC-3′ and Sequencing primer 5’-GTATTGCTGAGGGCCTTGAA-3′. Forward 5’-GGTTCCTACAATGGCACAAG-3′ and Reverse 5’-ACGTTACAGGTGGGATCGATA-3′ were used for human *PDK3* PCR.

### Body width measurement

Age-synchronised animals 48 h post L4 stage were mounted on 3% (w/v) agar pads containing 10 μL of 100 mM levamisole and imaged using a Leica DMI3000B inverted microscope and a ProgRes CF^Cool^ Camera. All images were captured using a 10x objective such that the tip of the head and tail were clearly visible. Body width measurement were carried out using ImageJ software as previously described (49).

### Aldicarb and levamisole assay

NGM plates containing 1 mM Aldicarb (Sigma-Aldrich) were poured the day before the experiment. On the morning of the experiment, 20 μL of concentrated OP50 was seeded in the middle of the plate and was allowed to air dry for 1–2 h. Day 1 adult animals were transferred to the aldicarb assay plate and scored for paralysis every 10 min for a period of 160 min. NGM plates containing 0.2 mM levamisole (Tetramisole hydrochloride, Sigma-Aldrich) were freshly prepared on the day of the experiment and air-dried for 1 h. Concentrated OP50 (20 μL) was then seeded in the middle of the plate and air dried for another hour. Day 1 animals were transferred to the NGM plates containing levamisole and scored for paralysis every 10 min for a period of 160 min. For both the aldicarb and levamisole assay, an animal was scored as paralysed by its unresponsiveness/inability to move when prodded with a metal wire twice in the head and the tail (30,50).

### Non-quantitative PCR based mitochondrial DNA copy number assay

PCR based methods determining mitochondrial DNA copy number in multiple species including *C. elegans* were used as previously described (16). Day 1 and day 4 old worms were used for mitochondrial DNA copy number quantification. Worm lysates were prepared using the *C. elegans* Lysis/DNA extraction kit (InVivo Biosystems) with some modifications. Six worms per age group were placed in a solution containing 5x Worm Lysis buffer A (2 μL), 10x Worm Lysis Buffer B (1 μL) and RNAse/DNAse free water (7 μL). The reaction mix was incubated at 75°C for 5 min followed by 10 min at 95°C. Following incubation, 80 μL of RNAse/DNAse free water was added. Worm lysate (5 μL) was used for amplification of mitochondrial DNA using the GoTaq Flexi kit (Promega). PCR amplification of mitochondrial DNA was performed using the following primers: mitochondrial DNA Forward: 5′- CACACCGGTGAGGTC TTTGGTTC-3′ and mitochondrial DNA Reverse: 5’-TGTCCTCAAGGCTACCACCTTCTTCA-3′. In this method, PCR products were used for quantification of mitochondrial DNA copy number. The generation of single band mitochondrial PCR amplicons was confirmed by size fractionating PCR products on a 1.5% (w/v) agarose in 1X TAE running buffer (Supplementary Material, Fig. S1). Prior to quantification, the MinElute PCR purification kit (Qiagen) was used to remove primers present in the PCR product.

The QuantiFluor ONE dsDNA System (Promega) was used for DNA quantification. A DNA standard curve was prepared using the control lambda DNA provided with the kit and the quantification was performed according to manufacturer’s instructions. Quantiflour DNA dye (200 μL) was mixed with a DNA sample of known concentration (1 μL) and samples of unknown concentration (1 μL) and incubated at room temperature for 5 min. Following incubation, the luminescence was measured using an Enspire Multimode Plate Reader (PerkinElmer) at an excitation wavelength of 504 nm and emission wavelength of 531 nm.

### Paraquat induced oxidative stress response assay

An oxidative stress response assay was performed using Paraquat dichloride hydrate (Sigma-Aldrich). The assay was carried out in a 24 well plate containing 0.2 M paraquat solution (250 μL). Age synchronised L4 stage animals were added to each well and scored for paralysis every hour for a period of 3 h. Paralysis following treatment with paraquat was scored based on body morphology as previously described (20).

### Live imaging for quantifying neurodegeneration

Age synchronised day 1, 4 and 8 animals (*oxIs12*, *hPDK3^WT^* and *hPDK3^R158H^*) were anesthetised using 100 mM levamisole and mounted on a 3% agar pad and imaged using a Leica DMI3000B inverted microscope and a ProgRes CF^Cool^ Camera. A 10x objective and GFP filter were used to capture the images of animals expressing GFP in the GABAergic neurons. The exposure time for day 1 animals was 250 ms and a 15 ms exposure time was used for day 4 and 8 animals to reduce the background intensity observed in the animal’s gut. Scoring for the neurodegeneration phenotype was carried out as previously described (51). GFP expression along the ventral nerve cord (VNC) of normal animals was continuous, while animals showing signs of axon degeneration exhibited GFP expression that was interrupted by blebbing or beading of the axon (ventral nerve cord) that eventually led to complete loss of the axon and/or neuron (Fig. 5A). Statistical analysis of the neurodegeneration quantification was carried out as previously described (52).

### Locomotion assays

Day 4 animals were used for the thrashing and body bend assays. On the day of the thrashing assay, animals were transferred to an unseeded NGM plate and allowed to crawl freely for 1 h to remove any bacteria attached to the body. For the thrashing assay, a single animal was transferred to a 30 mm plate containing NGM medium (3 mL). M9 buffer (1 mL) was added to the plate and the animal was allowed to swim for 1 min. Following acclimation, the video of the animal swimming was captured for 1 min using Leica M165FC microscope and Leica DFC3000G camera. The captured video was analysed manually to calculate the number of thrashes per min. The movement of the worm’s head and tail to the same side was counted as one thrash as previously described (47). For the body bend assays, a single day 4 worm was allowed to move freely, for 5 min on a 30 mm NGM plate. Following acclimation, a 3 min video was captured. Videos were analysed manually to calculate the number of body bends over the 3 min period. The maximum bend of the worm just behind the pharynx during the sinusoidal locomotion in the forward direction was counted as one body bend as previously described (22). The reverse locomotion of the animal was not included in the analysis.

### ATP quantification assay

The method used for ATP quantification was adapted from a previous study (53). 1500 to 3000 age synchronised day 1 animals were collected and washed three times using autoclaved Milli-Q water. After the final wash, animals were resuspended in autoclaved Milli-Q water (120 μL), snap frozen using liquid nitrogen and stored at −80°C. At the time of use, frozen tubes were removed from −80°C and thawed on ice. The freeze–thaw cycle was repeated three times followed by incubation at 99°C for 15 min and on ice for 5 min. The samples were centrifuged at 14800 *x g* for 10 min at 4°C. The supernatant (20 μL) was used for protein quantification using the Pierce BCA protein assay kit (ThermoScientific), with the remaining lysate being used for cellular ATP quantification using the ATPlite assay kit (PerkinElmer) as previously described (9). Luminescence detection and absorption to determine cellular ATP levels and protein levels was on an Enspire Multimode Plate Reader (PerkinElmer) as per manufacturer’s instruction.

### Statistical analysis

Prism software was used for the statistical analysis. One-way ANOVA and Tukey’s multiple correction test was used to calculate adjusted p-values for all the datasets unless mentioned otherwise. Corrected p-value < 0.05 was regarded as significant.

## Supplementary Material

DCA_Treatment_ddab228

HMG-2021-CE-00275_Narayanan_Supplementary_information_ddab228

Supplementary_Figure_1_ddab228

Supplementary_Figure_2_ddab228
