# Supplementary figures and images for: Charcot–Marie–tooth disease causing mutation (p.R158H) in pyruvate dehydrogenase kinase 3 (PDK3) affects synaptic transmission, ATP production and causes neurodegeneration in a CMTX6 C. elegans model
Source: Hum Mol Genet. 2021 Aug 13;31(1):133–45. doi: 10.1093/hmg/ddab228 (PMC8682796; doi:10.1093/hmg/ddab228)

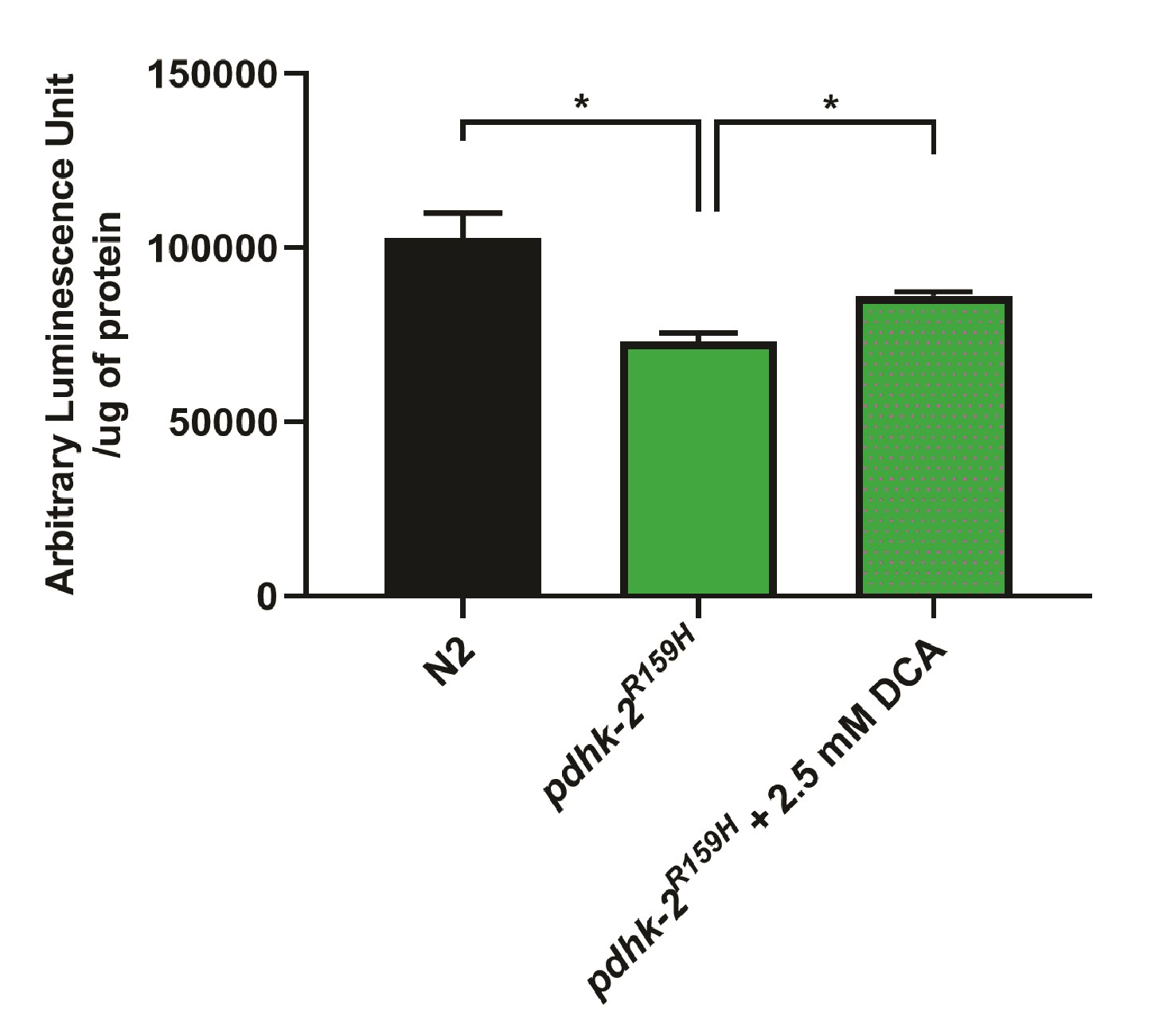

Supplement: DCA_Treatment_ddab228 [file dca_treatment_ddab228.jpeg]

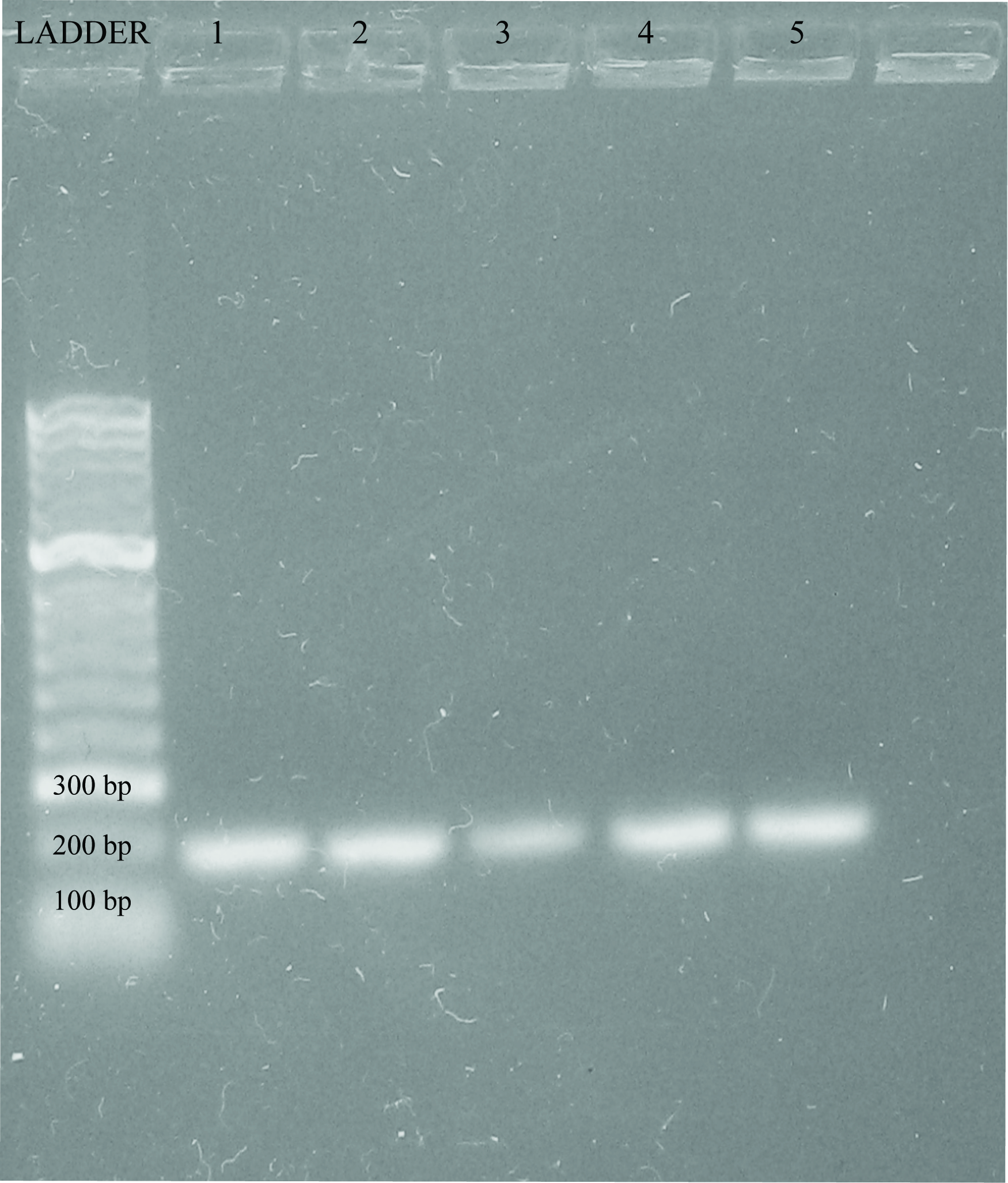

Supplement: Supplementary_Figure_1_ddab228 [file supplementary_figure_1_ddab228.zip › Supplementary_Figure_1_ddab228.tif]

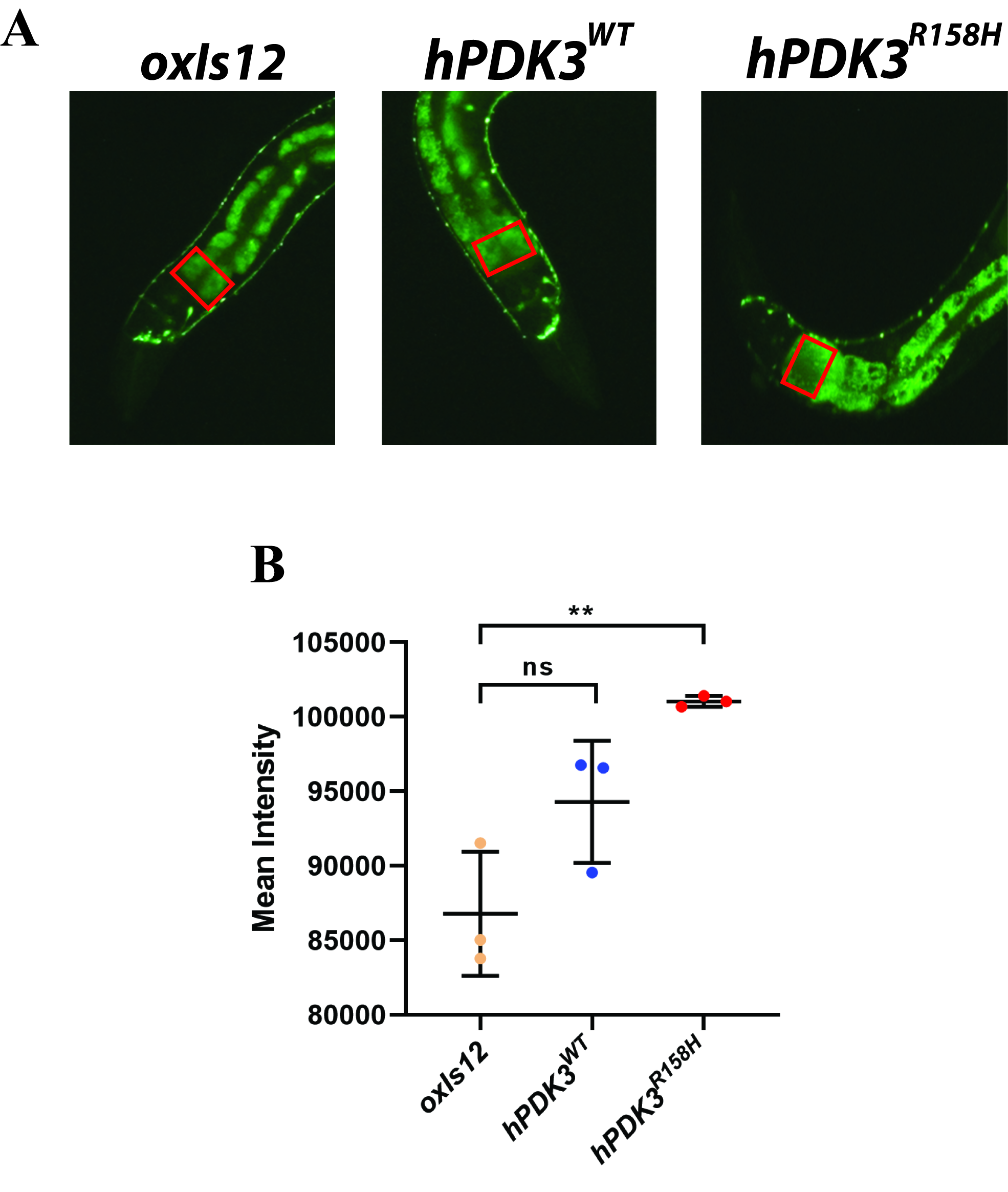

Supplement: Supplementary_Figure_2_ddab228 [file supplementary_figure_2_ddab228.zip › Supplementary_Figure_2_ddab228.tif]
