# Supplementary material for: Charcot–Marie–tooth disease causing mutation (p.R158H) in pyruvate dehydrogenase kinase 3 (PDK3) affects synaptic transmission, ATP production and causes neurodegeneration in a CMTX6 C. elegans model
Source: Hum Mol Genet. 2021 Aug 13;31(1):133–45. doi: 10.1093/hmg/ddab228 (PMC8682796; doi:10.1093/hmg/ddab228)
Supplement: HMG-2021-CE-00275_Narayanan_Supplementary_information_ddab228 [file hmg-2021-ce-00275_narayanan_supplementary_information_ddab228.docx]

**Generation of knock-in and overexpression *C. elegans* strains**

**CRSIPR-Cas9 generation of *pdhk-2^R159H^*:** We outsourced the generation of CRIPSR-cas9 mediated genetic modifications to SunyBiotech Pty Ltd. Below are the details of upstream and downstream flanking sequences in N2 and *pdhk-2^R159H^* respectively. The sequence change from AGA in wild type to CAC in *pdhk-2^R159H^* animals is highlighted in red. A synonymous mutation in the upstream of CAC change in the knock-in animal is labelled in blue. In-house bioinformatics tools developed by SunyBiotech Pty Ltd based on previously published methods (1, 2) was used for sgRNA targeting site selection and off-target site exclusion.

> N2

TGGCGAATATTATGCAGGAATTCACACTTCTTCCACCAAAGCTTCTTCAAATGCCAAGTTCAAAAATGGTATCAAATTGGTATGCTGAGAGTTTCGAGGATCTTCTTTTGTTCGAGGCATCTGATTCATCTCCAGAGCAAGTTGCAAGgtaagagaaaaaattgaatacctaatttacaaaaaaaaacttgattttttggttttaaaagtttagaaatacatttttaaaatattaatgttaatttatgtttttcagATTCAATGATCAGTTAACAGTCGTACTGAAAAGACACGCACATGTCGTCGAAACAATG**GCT**GAAGGATTGATTGAACTTCGAGAATCGGATGGAGTAGATATTGCAAGTGAAAAAGGAATTCAATATTTC**TTGGATCGA**TTTTACATC**AACAGA**ATTTCCATTCGTATGCTTCAAAATCAACATCTGgtaagttattttaaaaattttaatattttcaaataaaacattttttccagGTTGTATTCGGAAATGTTCTCCCAGAAAGTCCTCGTCATGTTGGATGTATTGATCCTGCCTGTGATGTGGAAAGTGTAGTGTACGATGCTTTTGAAAATGCTCGgtgagtgcaagttgatgataaaattgcctagaaattaaagaaataagccgataaaagtccaaagtttcaccagaaaatgttctaaaattttttttttgagattgatgttcaaggccctcagcaataccttccat

>*pdhk-2^R159H^*

TGGCGAATATTATGCAGGAATTCACACTTCTTCCACCAAAGCTTCTTCAAATGCCAAGTTCAAAAATGGTATCAAATTGGTATGCTGAGAGTTTCGAGGATCTTCTTTTGTTCGAGGCATCTGATTCATCTCCAGAGCAAGTTGCAAGgtaagagaaaaaattgaatacctaatttacaaaaaaaaacttgattttttggttttaaaagtttagaaatacatttttaaaatattaatgttaatttatgtttttcagATTCAATGATCAGTTAACAGTCGTACTGAAAAGACACGCACATGTCGTCGAAACAATG**GCA**GAAGGATTGATTGAACTTCGAGAATCGGATGGAGTAGATATTGCAAGTGAAAAAGGAATTCAATATTTC**TTAGACCGG**TTTTACATC**AATCAC**ATTTCCATTCGTATGCTTCAAAATCAACATCTGgtaagttattttaaaaattttaatattttcaaataaaacattttttccagGTTGTATTCGGAAATGTTCTCCCAGAAAGTCCTCGTCATGTTGGATGTATTGATCCTGCCTGTGATGTGGAAAGTGTAGTGTACGATGCTTTTGAAAATGCTCGgtgagtgcaagttgatgataaaattgcctagaaattaaagaaataagccgataaaagtccaaagtttcaccagaaaatgttctaaaattttttttttgagattgatgttcaaggccctcagcaataccttccat

**Generation of overexpression *C. elegans* model of CMTX6:** Gateway cloning (Invitrogen) method was used for generation of stable *C. elegans* transgenics that overexpress human wild type (*hPDK3^WT^*) and mutant PDK3 (*hPDK3^R158H^*). The coding sequence of human PDK3 was amplified from the cDNA library using primers that contain the attB1 and attB2 recombination sequences. The amplified cDNA was first recombined with the donor vector pDONR221, which was used as template to generate mutant PDK3 using site-directed mutagenesis kit (QuickChange II, Agilent). The entry vector containing human WT R158H cDNA was recombined with gateway compatible pPD157.60 vector containing the *unc-25* promoter sequences to form the expression plasmids. UNC-25, a glutamic acid decarboxylase is localized to axons and synapse and play a major role in synaptic transmission (3). A plasmid mini prep kit (Qiagen) was used for scaling up the concentration of expression plasmids. Cloning into pPD157.60 was confirmed by genotyping with *BsrGI* restriction digests of the constructs. *C. elegans* transgenics were generated by microinjecting a cocktail of expression plasmids containing the *unc-25* promoter and human PDK3 cDNA (WT or R158H) at a concentration of 10 ng/µL and plasmid PCFJ90 at a final concentration of 5 ng/µL. PCFJ90 plasmid drives mCherry expression in the pharynx of *C. elegans*, which allows easy screening of transgenic animals under fluorescent microscope. The human transgenes were injected into EG1285 animals carrying the *oxIs12[unc-47p::GFP + lin-15(+)]* transgene to create extra-chromosomal arrays. UNC-47, an ortholog of human solute carrier family 32 (SLC32A1) is a vesicular GABA transporter involved in the uptake of GABA into the synaptic vesicles (4).

To isolate transgenic strains containing extra-chromosomal arrays, 2 days post injection 20 F_1_ mcherry positive animals were separated onto individual NGM plates and maintained at 22°C. 2 to 3 days post isolation, F2 progenies of the isolated lines were analysed for the presence of mcherry positive progenies. 3 lines from the F_2_ progeny that show stable inheritance of the extra-chromosomal array and displaying similar intensities of the mcherry marker were separated and maintained at 22°C. The 3 separated lines were scored for the brightness of mcherry and % of the progeny carrying the marker for multiple generations to identify the strain that stable inherit the extra-chromosomal array for our experiments.

**Supplementary Figure 1.** Electrophoresis of purified PCR products used for mitochondrial DNA copy number quantification in Figure 4 on 1.5% (w/v) agarose in 1X TAE. A single amplicon (195 bp) of *C. elegans* mitochondrial NADH dehydrogenase subunit 5 gene was used for quantifying mitochondrial DNA copy number. Representative image of amplicon shown here was amplified from Day 1 old control and CMTX6 animals. LADDER - HyperLadder™ - 50 bp (Bioline), lane 1 – N2, lane 2 - *pdhk-2^R159H^,* lane 3 - *oxIs12*, lane 4 - *hPDK3^WT^* and lane5 - *hPDK3^R158H^*.

**Supplementary Figure 2.** (A) Intestinal autofluorescence serves as a proxy for energy storage in *C. elegans*. Representative images of day 8 old *oxIs12* and overexpression CMTX6 animals. First intestinal segment immediately following the pharynx (rectangle in red) was used for measuring intestinal autofluorescence. Image J was used for measuring fluorescence intensity. The site of measurement and the area of rectangle was similar for all strains used in this experiment. The measurement of fluorescence was limited to the area selected (rectangle in red). (B) *hPDK3^R158H^* animals displayed a significant increase in intensity levels when compared to *oxIs12* animals indicating that overexpression of mutant PDK3 led to a substantial increase in fat bodies when compared to wildtype. There was no significant difference in the intensity levels between *oxIs12* and *hPDK3^WT^* animals. Each data point refers to mean intensity of 10 animals per genotype. Three replicates per genotype were used for statistical analysis. ** adjusted p-value < 0.0012.

**Supplementary video 1.** Video of day 4 old *oxIs12* animal thrashing in M9 buffer post training (1 min).

**Supplementary video 2.** Video of day 4 old *hPDK3^R158H^* animal thrashing in 1 mL of M9 buffer following 1 min training.

**References**

1 Hsu, P.D., Scott, D.A., Weinstein, J.A., Ran, F.A., Konermann, S., Agarwala, V., Li, Y., Fine, E.J., Wu, X., Shalem, O. *et al.* (2013) DNA targeting specificity of RNA-guided Cas9 nucleases. *Nat Biotechnol*, **31**, 827-832.

2 Cho, S.W., Kim, S., Kim, Y., Kweon, J., Kim, H.S., Bae, S. and Kim, J.S. (2014) Analysis of off-target effects of CRISPR/Cas-derived RNA-guided endonucleases and nickases. *Genome Res*, **24**, 132-141.

3 Jin, Y.S., Jorgensen, E., Hartwieg, E. and Horvitz, H.R. (1999) The Caenorhabditis elegans gene unc-25 encodes glutamic acid decarboxylase and is required for synaptic transmission but not synaptic development. *J Neurosci*, **19**, 539-548.

4 McIntire, S.L., Reimer, R.J., Schuske, K., Edwards, R.H. and Jorgensen, E.M. (1997) Identification and characterization of the vesicular GABA transporter. *Nature*, **389**, 870-876.
